# Delivering the AAMC “Teaching for Quality” Program through a Community-Based GME Collaborative: Lessons Learned to Date

**DOI:** 10.51894/001c.6977

**Published:** 2018-09-26

**Authors:** Brandy Church, William Corser, Jonathan Rohrer, Kari Hortos, Angela Harrison

**Affiliations:** 1 Statewide Campus System, College of Osteopathic Medicine Michigan State University, East Lansing, MI

**Keywords:** graduate medical education, patient safety, quality improvement, scholarly activity

## Abstract

**CONTEXT:**

To address scholarly activity (SA) accreditation standards, the Michigan State University’s College of Osteopathic Medicine Statewide Campus System has offered the Association of American Medical Colleges’ (AAMC) Teaching for Quality Program for two cohorts of community-based faculty. The purpose of this paper was to describe the design and delivery of the customized program, the authors’ initial lessons learned, and their plans for further evaluation and dissemination.

**METHODS:**

The authors customized the program to overcome the barriers typically faced by community-based program faculty learners through a graduate medical education (GME) consortium model. This was the first time this program was delivered in this manner

**RESULTS:**

The authors’ initial cohort of 19 learners successfully developed 15 projects, with two pairs of learners collaborating on projects. The second cohort of 15 learners developed 11 projects, with one pair of learners collaborating. The authors present a series of principles for community-based GME leaders striving to develop SA projects in their respective GME environments.

**CONCLUSIONS:**

The “consortium advantage” derived from entities such as the SCS may prove integral to efficiently coordinating SA project resources and knowledge across diverse GME systems.

## INTRODUCTION

During recent years, graduate medical education (GME) in the U.S. has continued to experience substantial changes, most notably moving to a single accreditation system. Under this new system, graduates of both allopathic and osteopathic medical schools complete residency and/or fellowship training in programs accredited by the Accreditation Council for Graduate Medical Education (ACGME).^1^ All resident physicians and GME faculty are required to meet common standards and requirements as outlined in the ACMGE’s Next Accreditation System (NAS).^1,2^

Many community-based residency programs have experienced considerable challenges meeting these new ACGME requirements, particularly those related to increased faculty and residents scholarly activity (SA) project expectations and compliance with the Clinical Learning Environment Review (CLER).^3–6^ Community-based GME officials may be especially challenged meeting accreditation standards due to barriers including: 1) lack of time, 2) inadequate training and experience, and 3) lack of resources and knowledge required to complete SA projects and disseminate results.^7–12^

To address SA accreditation standards, the Statewide Campus System (SCS) at Michigan State University’s College of Osteopathic Medicine (MSUCOM)^13^ has offered the Association of American Medical Colleges’ (AAMC) Teaching for Quality (Te4Q) Program for two cohorts of community-based faculty.^14^ SCS customized the program to overcome the barriers typically faced by community-based program faculty by training cohorts of learners through a consortium model. This was the first time this program had been delivered in this manner.

The purpose of this paper is to describe the design and delivery of the program, the authors’ initial lessons learned, and their plans for further evaluation and dissemination. The authors will conclude with a series of general principles for GME leaders striving to develop projects at their respective SA environments.

## METHODS

### Setting

The SCS was founded in 1989 as a statewide consortium to improve the quality of Michigan osteopathic GME.^13^ Today, the SCS represents 37 community-based hospitals, 7 federally qualified health centers, and 176 residency programs accredited by the ACGME and/or the American Osteopathic Association (AOA). The consortium is charged with serving the GME needs of residency designated institutional officers, directors of medical education, program directors and over 1,900 residents and fellows.

### Program Planning

The Te4Q^14^ program is a multi-faceted faculty development program designed to train faculty learners to teach effective quality improvement and patient safety (QIPS) principles to medical students, residents, and other clinicians. The AAMC initially (2013) designed the program to train up to 30 faculty in a single institution over a 15-to-18 month timeframe to:

- Identify a gap in their QIPS education;
- Design a feasible QIPS project to address that gap;
- Conduct the project and assess its impact; and
- Produce a SA poster, article, or presentation concerning their project results.

This overall training program sequence is illustrated in Figure 1.

**Figure:**
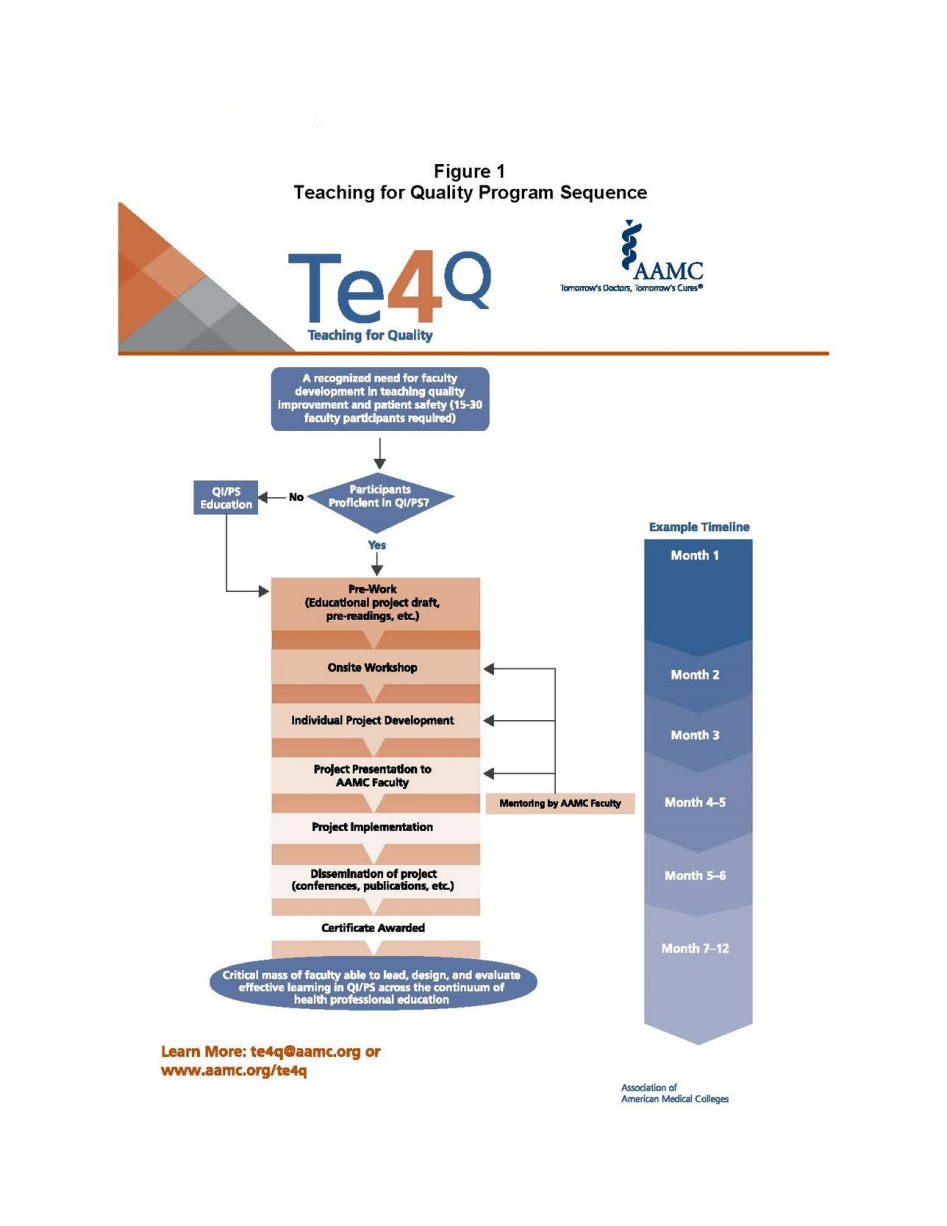
Figure 1 Teaching for Quality Program Sequence

During the original planning process, the SCS Office of Faculty Development recognized that program implementation through a consortium would present unique challenges (e.g. more complex cross-system communications, GME program variations, etc.) for both SCS “coaches” and faculty learners. Additionally, the authors recognized that the program would need to be customized to overcome the barriers typically faced by community-based clinicians conducting SA projects. Therefore, they proposed the following modifications to the delivery of the program to central AAMC office faculty who were especially supportive:

### Barrier #1: Lack of time

**Program resource identification.** The authors customized the Te4Q^14^ program during the following phases: preparation for participation, a 1.5-day project planning workshop, project design, implementation, results, analysis, and dissemination. Resources were then identified to help maximize learners’ efficiency progressing through each SA project phase. Project completion time estimates were provided, and this information was incorporated into all promotional program materials.

**Articulation of program time commitment.** The SCS leadership presented to its Education Standards Committee and Governing Board a detailed timeline, complete with monthly time estimates to which learners would need to commit. As both committees were comprised of leadership from each member hospital, this step was essential to ensure that learners’ institutional leaders would understand and be supportive of the commitment required for their faculty to complete the program. Program applicants were then required to complete a four-page learning contract, committing to the program time expectations.

### Barrier #2: Inadequate training and experience

**Common QIPS knowledge base.** Acknowledging that learners would attend this program with a variety of pre-program experiences, the authors devised a plan to establish a common QIPS vocabulary for program communications. Learners were encouraged to complete two Institute for Healthcare Improvement (IHI)^15^ modules concerning QIPS and read an article concerning CLER^3^ in addition to the required pre-workshop assignments. Learners were also encouraged to watch additional IHI modules and attend the SCS-sponsored workshop to at least “brush up” on QIPS content prior to starting their SA project planning. A library of over 300 publicly-accessible project support materials (i.e. voiced-over PowerPoint modules regarding feasible project design, IRB applications, data set creation, analytic techniques, project tip sheets and templates, pertinent GME articles, etc.) files were made available and posted on the program course website.

**SCS faculty coaches.** The SCS Office of Faculty Development quickly recognized from learners’ pre-workshop self-assessment statements that most learners had never developed a prior SA project. The authors therefore identified themselves to serve as learner coaches for different projects based on the project focus and the coach’s areas of expertise.

**Narrowed project focus.** SCS required program learners to select one CLER^3^ pathway focus area from a list of fifteen that would comprise their project focus. Learners then designed a project with their coaches within that domain that was also in alignment with their individual health system’s priorities. By narrowing the scope of program projects in this manner, the authors could help: 1) facilitate project feasibility, 2) learners utilize available SCS resources without overextending assigned coaches, and 3) create learner project clusters for possible SA project collaboration.^16–21^

### Barrier #3: Lack of institutional resources

**Communication and resource accessibility.** The authors worked to help ensure that learners would each have: 1) ready access to resources and materials for future projects, 2) a place to archive important project-related documents, and 3) a feasible mechanism to communicate with other learners and coaches. We therefore created an online course using Desire2Learn^22^ course management software.

This online course site became a repository of recent articles concerning SA in GME settings, resources related to IRB application and project design, project timeline and deadline templates, discussion boards for frequently asked questions, and drop boxes for important project documentation. The drop boxes also enabled coaches to offer ongoing guidance, consultation, and feedback based on what learners had submitted.

### Learner recruitment

The recruitment of Te4Q^14^ program applicants included promotional materials containing information about how participation would help them meet NAS and SA standards. Additional proposed reasons to apply included that: a) completed SA projects would improve GME education in QIPS and awareness of system errors; b) provide learners with SA skills for themselves and their residents; and c) result in systemic improvement in one specific CLER^3^ pathway. We successfully enrolled a total of 19 participants from 13 different SCS member systems in our first 2015-2016 cohort and 15 participants from 10 different SCS member systems in our second 2017-2018 cohort.

We have concluded that the overall process of promoting the program as an appealing GME investment primarily involved two simultaneous processes: a) obtaining “buy-in” from multiple system and residency program stakeholders, and b) adequately customizing the program to prove more feasible/appealing to potential community-based GME learners.

### Program Implementation

In January 2015, applicants were notified of their program selection and provided a packet of pre-workshop assignments with completion deadlines and the contact information of their assigned coach. AAMC faculty primarily taught the workshop in March. Learners then worked with their coaches to refine their project, evaluate project feasibility, and prepare appropriate IRB applications.

A follow-up webinar occurred that August with AAMC faculty, SCS coaches, and learners to provide a 10-minute status report on their projects. At the end of the program in May of the next year, learners disseminated their SA project findings at a regional poster day to earn completion certificates from both the AAMC and MSU.

## RESULTS

Our initial 2015-2016 cohort of 19 learners developed 15 projects, with two pairs of community-based faculty collaborating on two separate projects. Two learners withdrew from the program, one leaving the area for another GME position. Our second 2017-2018 cohort of 15 learners developed 11 projects, with one pair of participants opting to collaborate. Three learners withdrew from the program due to positional changes or competing GME position demands.

To date, about 50% of program projects have been specifically oriented to testing the implementation, delivery, and evaluation of QIPS curricula and content for either residents and/or faculty. The remaining projects addressed specific aspects of healthcare delivery processes such as surgical suite waiting times, postoperative patient follow-up calls, opioid prescribing patterns, cross-shift resident handoffs, timely reporting of critical patient lab results, assessing patients for severe sepsis, and implementing a series of group visits for patients with complex diabetes management needs.

Two projects involved education of residents concerning osteopathic manipulative medicine treatments. Those SA projects involving any protected patient health information were designed to have system Quality Improvement department personnel de-identify patient data to avoid HIPAA-related infringements^23^ and facilitate IRB approval.

## DISCUSSION

We have identified the following principles for GME settings:

1. Compiling resources and streamlining project design processes can reduce learner time commitment and increase their SA project engagement.

Since community-based faculty are so often busy with patient care demands, prospective QIPS projects can be perceived as “just one more thing to be done”.^7,9,10,12,24^ Still, our Te4Q learners generally indicated that their project planning was made more manageable by proactively planning their project schedules based on provided timeline templates. Several learners also stated that the resources SCS coaches had provided them helped them save time. Some learners placed positive comments of other posted online resources.

In hindsight, it appears that those learners who utilized available program-related resources tended to have smoother project design experiences. It was, however, also especially evident that sizable variations existed across systems (e.g. degree of QI department capability, complexity of IRB application reviews, accessibility of data-capable support personnel, potential resistance from other GME faculty) which either facilitated or impeded successful project completion.^8,17,24–27^

The SCS Office of Faculty Development also reviewed overall differences in project design needs between primary care and specialist providers, the extent to which different learners wanted to obtain (or avoid using) protected patient health data. The authors have concluded that regularly assessing such differences across learners’ healthcare systems will be helpful for future Te4Q cohorts. Clearly, some learners preferred to be left alone after being provided a “critical mass” of project support materials, while others preferred to exchange project updates or engage in ongoing problem-solving discussions with coaches and colleagues.^11,24^

2. Helping learners attain a solid QIPS knowledge base may make them more confident and capable of completing SA projects

Most learners stated that the assigned pre-workshop IHI^15^ modules and readings enhanced their understanding of QIPS concepts and project design principles. Although the QIPS workshop has probably been effective for most learners, the actual outcomes derived from such intensive content-laden events may be less helpful for more novice learners. Since different types of SA workshop/“boot camp” approaches exist,^20,28^ the SCS Office of Faculty Development is currently revising the workshop curriculum for future Te4Q cohorts.

3. Providing assigned coaches with compatible project-related skills can serve to keep learner SA projects moving forward.

The provision of coaches with compatible osteopathic provider, educational specialist, and project design expertise appeared to be critical for many learners during their early project planning. Similar to other settings, several program learners indicated that they appreciated most coach feedback, as well as the accountability they felt to complete project developments to provide periodic updates for their coaches.^18,29–32^

## CONCLUSIONS

This paper summarizes the authors’ experiences of delivering the AAMC Te4Q^14^ program to two cohorts of community-based faculty through an established GME consortium. Since both community and university-based residency program officials are now required to meet increasingly rigorous SA standards, the principles outlined in this paper may prove generalizable to the complex SA challenges of GME officials across the nation.

It will very likely take several additional cohorts of learners for the authors to determine the sustainability of programs such as Te4Q^14^ to enable diverse community-based GME leaders to: a) improve their capacity for SA projects, b) implement meaningful SA project supports, and c) incrementally refine key processes to sustain momentum gained from initial SA projects.^23,31–33^

Several groups have suggested that the “consortium advantage” from such entities as the SCS may prove integral to more efficiently sharing SA resources and capitalizing on project-related expertise across diverse GME settings.^18,25,30,31^ Ideally, these paper conclusions will contribute to the development of more innovative approaches for the thousands of community-based GME programs now held to SA expectations.^34,35^

### Conflict of Interest

The authors declare no conflict of interest.
